# Effects of Eccentric Speed during Front Squat Conditioning Activity on Post-activation Performance Enhancement of Hip and Thigh Muscles

**DOI:** 10.5114/jhk/183917

**Published:** 2024-04-15

**Authors:** Petr Stastny, Dominik Kolinger, Anna Pisz, Michał Wilk, Jan Petruzela, Michał Krzysztofik

**Affiliations:** 1 Department of Sport Games, Faculty of Physical Education and Sport, Charles University in Prague, Prague, Czech Republic.; 2 Institute of Sports Science, The Jerzy Kukuczka Academy of Physical Education, Katowice, Poland.

**Keywords:** post-activation potentiation, complex training, power training, isokinetic, handball

## Abstract

The phenomenon of post-activation performance enhancement plays an unidentified role in movement eccentric speed and individual muscle group responses. Therefore, this study aimed to determine whether the loaded front squat (FSq) speed of the eccentric phase would influence the post-activation performance enhancement effect and whether the FSq would elicit similar performance enhancement of knee flexion, knee extension, hip flexion, and hip extension muscles. Twenty resistance-trained handball players performed the FSq under maximum eccentric-concentric speed and 2-s eccentric speed (only the eccentric phase performed), while pre- and post-front squat countermovement jump, knee, and hip isokinetic flexion/extension performance were tested. The FSq conditioning activity was performed in a single set of three repetitions with either 90% (maximum eccentric-concentric speed) or 120% (2-s eccentric speed) of one repetition maximum, and post-performance was measured 4–12 min after the FSq. Athletes randomly changed the FSq eccentric speed and tested the hip or knee isokinetic flexion/extension strength at 180°/s. ANOVA showed that the rate of force development during the jump increased (Cohen d = 0.59–0.77) with no differences between 2-s eccentric and maximum speed eccentric protocols. Isokinetic strength increased after the 2-s eccentric FSq in hip extension (d = 0.76–0.86), knee flexion (d = 0.74–0.88), and hip flexion (d = 0.82), with no differences in knee extension strength. After maximum eccentric-concentric speed, isokinetic strength increased in hip extension (d = 1.25). In conclusion, the FSq conditioning activity enhances hip extensors' performance more than knee extensors' performance. Different eccentric types of muscle action during a conditioning activity alter the level of local muscle enhancement.

## Introduction

One of the effective ways to increase an upward and rightward force-velocity relationship is the use of a loaded conditioning activity (CA) before a power exercise to evoke acute post-activation performance enhancement (PAPE) (Blazevich and Babault, 2019; Blecharz et al., 2022; Cormier et al., 2022; Trybulski et al., 2022; Wolska et al., 2023) or increased competition performance (Bogdanis et al., 2014; Chua et al., 2021). Some general mechanisms such as changes in muscle temperature, cellular water content, muscle activation, alterations in the motor pattern, and post-activation potentiation effect have been identified as PAPE causes (Blazevich and Babault, 2019; Xenofondos et al., 2023). However, there is no identification of the contribution of particular variables of PAPE, like the optimal contraction speed of involved muscle groups. Therefore, the understanding of possible motor pattern changes and the role of CA speed should be investigated to determine the importance of exercise selection to elicit PAPE.

In the vast choices of CA patterns, squats are frequently used to elicit the PAPE effect in sprinting and jumping (Krzysztofik et al., 2023a–c), where a specific type of the motor pattern influences PAPE magnitude. For example the power clean elicited a higher PAPE effect in a sprint than a loaded squat (Seitzet al., 2014), and this difference was hypothetically explained by greater force production at high-velocity movements and more foot strike activation (Seitz et al., 2014) during the power clean, as they are not present in the squat. Although this explanation seems relevant, it has not been proved by empirical studies and does not explain all differences between the squat and power clean muscle patterns, such as the types of muscle action. Furthermore, it has been shown that the half squat CA in a concentric ballistic action is more effective than a non-ballistic one (Suchomel et al., 2016) and more effective when performed in isometric than concentric and eccentric action (Bogdanis et al., 2014). At the same time, the highest CA effect is often reported in plyometric activities (Seitz and Haff, 2016). These inconsistencies indicate that eccentric action and its velocity are significant contributing factors to the squat potentiation effect, where eccentric overloading enhances concentric velocity during the front squat (FSq) and accentuated eccentric loading performed from a drop jump increases countermovement jump (CMJ) performance (Bridgeman et al., 2017; Hughes et al., 2016) more than the traditional squat (Hughes et al., 2016) or the eccentric leg press exercise at 105–125% of one repetition maximum (1RM) (Ong et al., 2016). Thus, there is evidence that eccentric action can elicit concentric PAPE, however, the role of eccentric speed remains unclear in the current literature.

The exercise selection of a CA is typically based on the kinetic chain similarity to the performance task, where the squat is considered highly similar to vertical jumps due to the same hip extension moment (Sugisaki et al., 2014). However, this similarity does not include the recruitment of agonist muscles, which differs between the back squat and plyometric squat jumps, and where their activation does not have to correspond with the joint kinetics (Sugisaki et al., 2014). Thus, differences in activated muscle patterns often explain the differences in the PAPE effect between small exercise modalities. For instance, a deep squat resulted in higher PAPE during the CMJ than a quarter squat, with a hypothetical explanation that the deep squat elicits more gluteus maximus activation (Esformes and Bampouras, 2013). It is known that squatting as a CA increases CMJ performance and prime mover muscles’ activity (Sotiropoulos et al., 2010). However, whether whole muscle patterns or specific prime movers get activated after squatting is questionable. A deep front squat may be more effective in activating the posterior hip extension muscle chain (Gullett et al., 2009) with lower knee compressive force than a back squat (Chamera et al., 2023; Gullett et al., 2009; Papla et al., 2022; Spieszny et al., 2022).

There is an unidentified role of eccentric speed during the FSq as CA in PAPE and unknown responses of individual muscle groups activated by the FSq CA, which cause PAPE during jumping performance. Therefore, this study investigated whether the loaded FSq speed of the eccentric phase would influence the PAPE effect and whether the FSq would elicit similar performance enhancement of knee flexion, knee extension, hip flexion, and hip extension. We hypothesized that fast eccentric speed would result in a higher PAPE effect, which would be similar among knee and hip muscles.

## Methods

### Experimental Approach to the Problem

**Figure 1:**
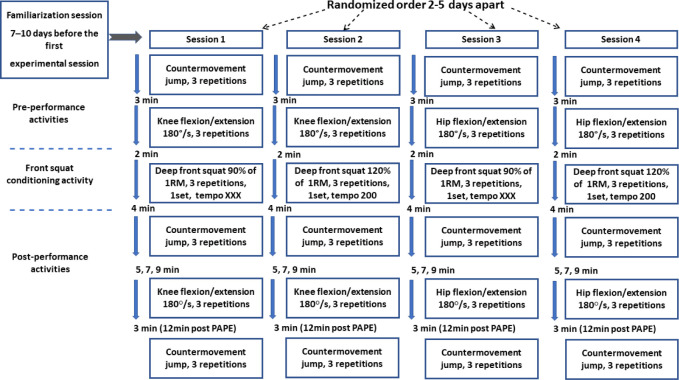
The timeline of front squat conditioning activities, the countermovement jump, and isokinetic testing during four experimental sessions.

This cross-sectional randomized research consisted of one familiarization session and four experimental sessions. The cross-sectional approach was used to describe the acute effect of different CAs which differed in the exercise tempo and eccentric loading. Two conditions of FSq movement speed were applied in four sessions, which differed in the measurement of particular muscle group PAPE. PAPE was observed in hip flexion/extension or knee flexion/extension (Figure 1) before and after the FSq to compare fatigue or potentiation effects individually for hip and knee muscles. Each experimental session included the CMJ performance to control the whole kinetic chain PAPE (Figure 1).

The familiarization session was completed 7–10 days before the first experimental session. Participants were familiarized with the experimental protocols and isokinetic testing and were measured for 1RM for the barbell FSq exercise. All experimental sessions used the FSq as the CA, and the countermovement jump (CMJ) and isokinetic strength of knee and hip flexion/extension as performance tests (Figure 1). The FSq was randomly performed under two conditions, in a 2-s eccentric tempo 2/0/0 (FSqE_2s_) with a load of 120% of 1RM and a fast eccentric-concentric tempo X/X/X (FSqE_X_) with with 90% of 1RM. A high intensity of 90% of 1RM was selected to be close to the eccentric load. The CMJ was performed in the same timeline during all four sessions: 5 min before the CA, 4 min, and 12 min after the CA. The isokinetic tests were performed during two different eccentric speed sessions for knee flexion/extension and two different eccentric sessions for hip extension/flexion with isokinetic measures of 3 min pre CA, and 5, 7, and 9 minutes post CA.

### Participants

The sample size was calculated a priori using G*power software (version 3.1.9.3, Dusseldorf, Germany) for repeated measures ANOVA within-between interaction with statistical power of 0.90, a level of significance of 0.05, and effect size *d* = 0.8, taking multiple studies reporting an effect size of 0.68 to 1.17 in PAPE during isokinetic testing (3–5). A minimum sample size of 14 individuals was obtained for two measures and of 10 individuals for four measures.

Sixteen (n = 16) healthy youth handball players (age 16.5 ± 1.2 years; body mass 79.8 ± 9.9 kg; body height 184 ± 6.5 cm; body fat content 10.3 ± 5.2 %; FSq 3RM 74.1 ± 19.1 kg, relative front-squat strength 1.00 ± 0.21 kg/body mass) of the first Czech national league in the U19 category participated in the experiment. The selection of youth handball players was based on the age sensitivity for power development (Lloyd and Oliver, 2012), which is the main quality of PAPE training. Moreover, handball players were familiar with all variations of jumping and exercising with high and eccentric loads.

Participants were selected based on the following inclusion criteria: resistance training experience (a minimum of two years), age (a minimum of 16 years), handball-specific training experience (a minimum of eight years), injury-free for the last three months. All participants were instructed to avoid resistance training 48 h before every session. Furthermore, all participants or their legal representatives gave their written consent to take part in the study. The study's research protocol was approved by the Ethics Committee of the Charles University, Faculty of Physical Education and Sport (approval code: EK267/2020; approval date: 06 November 2020).

### Procedures

To prepare participants for the study, considering their strength, they underwent four weeks of resistance pre-conditioning before the commencement of the study. This period was followed by a measurement of the deep barbell front-squat 1RM. The initial weight for the 1RM trial was self-selected; when the trial was successful, the weight was increased by 2.5–5 kg; when unsuccessful, it was decreased by 2.5–5 kg.

An attempt was only considered successful when the participant reached parallel to the bottom part of the deep squat position. After 1 RM determination, participants were familiarized with both squat protocols at different eccentric speeds (FSqE_2s_ with a load of 120% of 1RM and FSqE_X_ with 90% of 1RM), after which the CMJ was performed. Afterwards, participants were familiarized with isokinetic measures of knee and hip muscles. During both protocols, two spotters were securing the barbell and were lifting the barbell in the concentric action during the FSqE_2s_. During the FSqE_X_ with 90% of 1RM, participants were instructed to perform the movement as fast as possible, especially in the eccentric/concentric transition phase.

### Experimental Session

Each main session began with a non-specific warm-up consisting of 5 min of stationary cycling at moderate intensity, followed by bodyweight dynamic single-joint and multi-joint stretching and five countermovement jumps with subjective 70% effort. After a warm-up and a 3-min rest interval, the pre-test was performed in the form of three consecutive maximal CMJs or with instruction to jump as high as possible. The CMJ depth was self-selected, and one minute after the CMJ pre-test, the second pre-test was performed on an isokinetic dynamometer (IKD) either for the knee or the hip joint (depending on the selected protocol).

After 3 min of rest, participants performed a CA in the form of 3 sets of 3–4 repetitions of the barbell front-squat with loads corresponding to 60–90% of 1RM. The first set was performed at 60% of 1RM with four repetitions, and the second and third sets were performed at 90% or 120% of 1RM with three repetitions, respectively. The rest interval between each set lasted 3 min.

The measured variables after the CA (post-test) were the same as the pre-test variables and measurements took place in the following time sequence: at minutes 4 and 12 after the CA, the CMJ test was performed on the force plates; at minutes 5, 7, and 9, the post-test was performed on the isokinetic dynamometer. The two main sessions were separated by a minimum of 48 h and a maximum of a 7-day rest period. Participants were instructed to avoid heavy lower body strength training 48 h before experimental sessions.

### Isokinetic Dynamometry

Isokinetic measurements were performed at an angular velocity of 180°/s on an isokinetic dynamometer (Humac Norm, Stoughton, MA, USA) in the form of three flexions and three extensions in the concentric and eccentric muscle action modes to examine further the CA effects, which may manifest differently across muscle action types. The measured limb was weighed before each flexion and extension to separate the correct gravity from the final output values. Participants were verbally encouraged to perform their best throughout the measurement, and visual feedback on the force-position curve was provided.

For knee flexion and extension, participants were seated with a 90° angle at the knee and an 85° angle at the hips. The rotation of the lever and the knee of the dominant leg was set in a range of motion from 10° to 90°. During hip flexion and extension, participants were placed in a prone body position with their dominant lower limb thigh fixed with the lever arm and knees flexed to 90° along with the edge of the dynamometer seat. The rotation of the lever and the hip were set in a range of motion from 0° (fully extended) to 120° of flexion. Peak torque (PT) of the best repetition and the hamstring to quadriceps ratio were used for statistical analysis.

### Jumping Performance

Three CMJs were performed on two force plates (Hawking Dynamics, Westbrook, United States of America), each for one limb. The hands were placed on the hips to avoid using the upper limbs during the jump. Participants were instructed to jump as high as possible and come to a complete stop after each jump, and each CMJ started from a standing position. The depth of the countermovement was self-selected, as well as the width of the stance. The jump height was determined from the flight time impulse using the formula: Jump height = (take off velocity^2^ / (2 x g)). The braking phase was defined when the participant's center-of-mass velocity was still negative, but ascended toward 0 m/s. The breaking rate of force development was defined as the average slope of the vertical ground reaction force applied to the center of mass during the braking phase (McMahon et al., 2018).

### Statistical Analysis

Statistical data analysis was conducted using STATISTICA software (TIBCO, Palo Alto, CA, USA) with R software 3.2.1 integration (RStudio, PBC, Boston, MA, USA) at significance power of α = 0.05. The Shapiro-Wilk test was used for data distribution analysis, and the interrater intraclass correlation coefficient was used to check data reliability. The data were analyzed separately from each measurement and post values were analyzed as best post CA measure values. For normally distributed data, the two-way analysis of variance (ANOVA) for repeated measures followed by the Bonferroni post hoc test was used to analyze differences between pre- and post-CA measures and between the types of CA protocols (performance value x time pre/post measure x type of the protocol FSqE_2s_/FSqE_X_). Cohen's *d* with 95% confidential intervals (CI) was used to indicate the effect size of the difference between measurement time points (0.2–0.39 = small effect, 0.4–0.79 = medium effect, ≥ 0.8 = large effect).

## Results

The data obtained at all-time points did not violate normality, and intra-class correlation within the pre-post measures resulted in a coefficient of 0.8 and higher.

### Jump Performance

**Figure 2:**
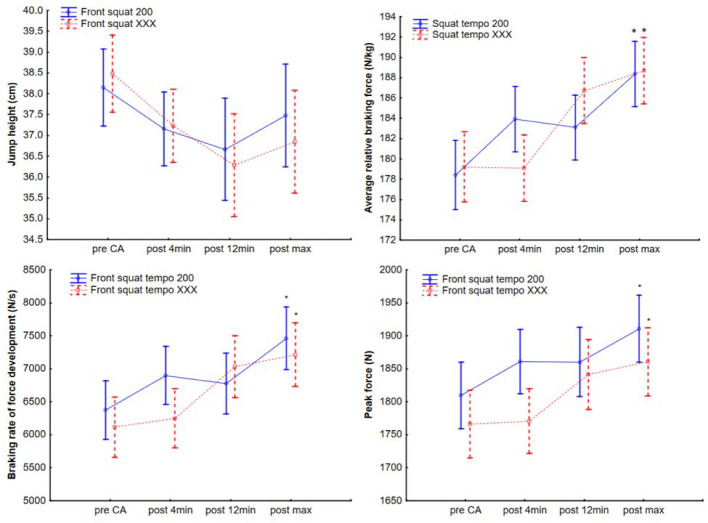
Jump performance before and after front squat conditioning activity at 200 and XXX tempo protocols *Significant difference to pre-CA; CA = conditioning activity

Repeated measures ANOVA showed no differences between pre- and post-jump height (F_1, 62_ = 0.82, *p* = 0.37, Figure 2, Table 1), but an increased rate of force development (RFD) (F_1, 62_ = 31.3, *p* < 0.001, Figure 2, Table 1), greater average relative braking force (F_1, 62_ = 28, *p* < 0.001, Figure 2, Table 1) and peak force (F_1, 62_ = 33.6, *p* < 0.001, Figure 2, Table 1). The Bonferroni post hoc test showed an increased RFD, greater average relative braking force, and peak force between the pre- and post-CA jump values with no differences in the CA protocol.

**Table 1:** Basic description of countermovement jump performance values before and after front squat conditioning activity within two different eccentric protocols.

Time / performance	CA protocol	Pre-front squat CA	4 min post CA	12 min post CA	Post max	Pre-Post max Cohen’s *d*
Jump height (cm)	FSqE_2s_	38.2 ± 5.1; 39.9–36.4	37.2 ± 4.7; 38.8–35.5	36.7 ± 4.9; 38.4–35	37.5 ± 4.9; 39.2–35.8	0.6
	FSqE_X_	38.5 ± 5.2; 40.3–36.7	37.2 ± 5.1; 39–35.5	36.3 ± 8.3; 39.2–33.4	36.8 ± 8.4; 39.7–34	0.28
Rate of force development (Ns)	FSqE_2s_	6376.4 ± 2627.8; 7286.9–5465.9	6896.9 ± 2698.4; 7831.8–5962	6776.7 ± 2494.5; 7640.9–5912.4	7461.1 ± 2773.2; 8422–6500.3*	0.71
	FSqE_X_	6108.6 ± 2297; 6904.4–5312.8	6234.4 ± 2135.3; 6974.2–5494.6	7035 ± 3641 7950–6120	7169 ± 2475.9 8026.9–6311.2*	0.69
Av. Relative braking force (N)	FSqE_2s_	178.4 ± 20.9; 185.6–171.2	183.9 ± 19.7; 190.7–177.1	183.1 ± 17.3; 189.1–177.1	188.4 ± 18; 194.6–182.1*	0.77
	FSqE_X_	179.1 ± 16.5; 184.8–173.4	179 ± 15.6; 184.4–173.6	186.7 ± 18.2; 193–180.4	188.3 ± 17.5; 194.4–182.3*	0.59
Peak force (N)	FSqE_2s_	1810 ± 286.5 1909.2–1710.7	1860.9 ± 301 1965.2–1756.6	1860.3 ± 297; 1963.2–1757.3	1910.8 ± 299.6; 2014.6–1807*	0.76
	FSqE_X_	1765.6 ± 269.8; 1859.1–1672.1	1770.2 ± 226.7; 1848.7–1691.6	1841.2 ± 285.4; 1859.1–1672.1	1857.4 ± 262.6; 1948.4–1766.4*	0.69

Reported values are mean ± standard deviation; ± 95% confidence interval; FSqE_2s_ = a protocol with a two-second eccentric front squat conditioning activity; FSqEX = a protocol with the front squat performed with maximum eccentric/concentric speed; CA = conditioning activity; * significant differences by ANOVA to the Pre-front squat CA at *p* < 0.001.

### Hip Joint Peak Torque Changes

**Figure 3:**
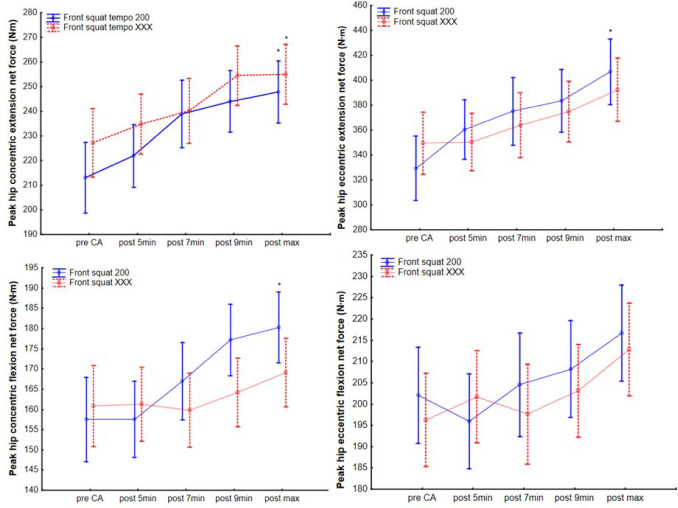
Hip joint net force (moment) before and after front squat conditioning activity at 200 and XXX tempo protocols. *Significant difference to pre-CA, CA = conditioning activity

The hip joint performance increased between pre- and the maximum post-CA values in peak net force during concentric hip extension (F_1, 30_ = 25.5, *p* < 0.001, Figure 3, Table 2) for both protocols, hip eccentric extension (F_1, 30_ =20.1, *p* < 0.001, Figure 3, Table 2) for the FSqE_2s_ protocol, and concentric hip flexion (F_1, 30_ =14.3, *p* = 0.001, Figure 3, Table 2) for the FSqE_2s_ protocol. No interaction was found between both eccentric protocols, and the only difference was in the post hoc test results under repeated measures values.

**Table 2:** Basic description of isokinetic performance at 180°/s before and after front squat conditioning activity within two different eccentric protocols.

Time / performance	CA protocol	Pre front squat CA	5 min post CA	7 min post CA	9 min post CA	Post max	Pre-Post max Cohen’s *d*
**Hip extension concentric (N•m)**	FSqE_2s_	223.2 ± 49.5; 247.5–199	228.4 ± 37.9; 247–209.9	242.4 ± 33.5; 258.8–225.9	250.9 ± 49.5; 268.4–233.3	255.5 ± 36.4; 273.3–237.7*	0.76
	FSqE_X_	225.9 ± 57.7; 253.6–198.1	234.8 ± 56.7; 262.5–207	239.6 ± 59.1; 268.5–210.7	253.6 ± 52.8; 279.4–227.7	254.1 ± 53.4; 280.2–227.9*	1.25
**Hip extension eccentric (N•m)**	FSqE_2s_	349.2 ± 101; 398.6–299.7	376 ± 110.3; 430–321.9	380.6 ± 91.3; 425.3–335.8	401.4 ± 91.7; 446.3–356.4	426.5 ± 95.6; 473.3–379.6*	0.86
	FSqE_X_	350.2 ± 95; 396.7–303.6	350.4 ± 75.3; 387.3–313.5	365.9 ± 99.7; 414.7–317	381.9 ± 98; 429.9–333.9	398.2 ± 102.8; 448.5–347.8	0.7
**Hip flexion concentric (N•m)**	FSqE_2s_	162.9 ± 32.9; 179–146.8	162.2 ± 32.5; 178.1–146.3	169.7 ± 27.3; 183.1–156.3	182.3 ± 27.4; 195.7–168.9	185 ± 28.2; 198.8–171.2*	0.82
	FSqE_X_	159.2 ± 41.5; 179.5–138.8	161.3 ± 38.6; 180.2–142.4	163.2 ± 41.3; 183.5–143	162 ± 36.9; 180.1–144	172 ± 36.4; 1189.8–154.1	0.48
**Hip flexion eccentric (N•m)**	FSqE_2s_	214.1 ± 43.8; 235.5–192.6	204.6 ± 45.3; 226.8–182.4	204.9 ± 34.5; 221.8–188	214.1 ± 43.8; 238.5–195.9	224.6 ± 42; 245.2–204	0.33
	FSqE_X_	196.4 ± 46; 218.9–173.8	201.7 ± 46.4; 224.4–178.9	200.7 ± 50.3; 225.4–176	204.2 ± 44.8; 226.1–182.2	214.9 ± 45.1; 237–192.8	0.67
**Knee extension concentric (N•m)**	FSqE_2s_	169.9 ± 35.2; 187.2–152.7	166.5 ± 30.3; 181.4–151.7	170.3 ± 32.3; 186.2- 154.5	165.3 ± 28.1; 179.1–151.6	173.4 ± 33.1; 189.6–157.2	0.16
	FSqE_X_	174.1 ± 28.7; 188.1–160	169.8 ± 32.2; 185.6–154.1	166.2 ± 28.4; 180.1–152.3	168.8 ± 29.3; 183.2–154.5	174.3 ± 30.9; 189.5–159.2	0.01
**Knee extension eccentric (N•m)**	FSqE_2s_	248.3 ± 54.4; 275–221.7	245.9 ± 59.4; 274.9–216.8	244.8 ± 55.6; 272.1–217.6	246.2 ± 51.7; 271.5–220.9	255.2 ± 56.7; 283–227.4	0.32
	FSqE_X_	255 ± 51.7; 208.3–229.7	241.1 ± 62; 271.6–210.8	236.1 ± 55.1; 263.1–209.1	238.2 ± 58.4; 266.8–209.6	254.2 ± 62.8; 285–223.5	0.03
**Knee flexion concentric (N•m)**	FSqE_2s_	100.3 ± 23.8; 112–88.7	101.3 ± 21.6; 111.9–90.7	105.3 ± 25.7; 117.9–92.8	107.2 ± 23.8; 119.8–94.6	109.5 ± 25.4; 122–97*	0.74
	FSqE_X_	99.1 ± 20.7; 109.2–89	99.8 ± 19.2; 109.2–90.3	102 ± 21.1; 112.3–91.6	102.6 ± 21; 112.9–92.4	106.6 ± 2-.5; 116.7–96.6	0.63
**Knee flexion eccentric (N•m)**	FSqE_2s_	173.4 ± 45.6; 195.7–151.1	174.9 ± 48.9; 198.9–151	180.9 ± 56.2; 208.5–153.4	180.6 ± 49.5; 204.9–156.3	188 ± 55.3; 215.1–160.9*	0.88
	FSqE_X_	166.3 ± 37.6; 184.7–147.8	163.5 ± 44.7; 185.4–141.6	163.2 ± 42.2; 183.9–142.6	166 ± 41.2; 186.2–145.8	174.8 ± 38.6; 193.8–55.9	0.57

Reported values are mean ± standard deviation; ± 95% confidence interval; FSqE_2s_ = a protocol with a two-second eccentric front squat conditioning activity; FSqE_X_ = a protocol with the front squat performed with maximum eccentric/concentric speed; CA = conditioning activity; * significantly different from the Pre-front squat CA.

### Knee Joint Peak Torque Changes

**Figure 4:**
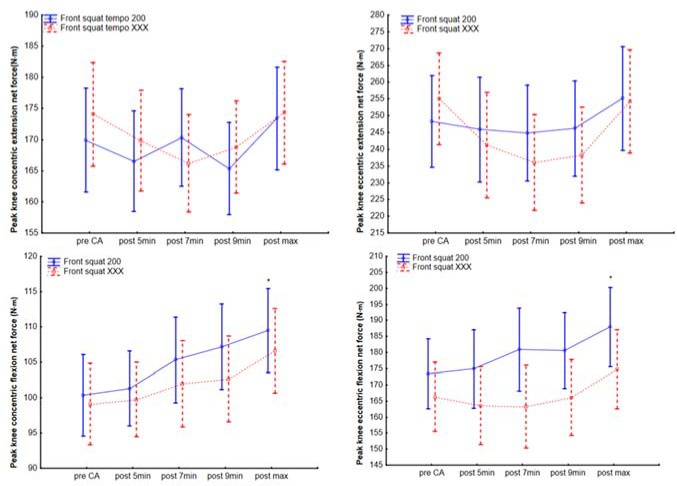
Knee joint net force (moment) before and after the front squat conditioning activity at the 200 and XXX tempo protocols. *Significant difference to pre-CA, CA = conditioning activity

The knee joint performance increased between pre- and the maximum post-CA values in peak net force during concentric knee flexion (F_1, 30_ = 15.2, *p* < 0.001, Figure 4, Table 2) for the FSqE_2s_ protocol and for eccentric knee flexion (F_1, 30_ = 16.9, *p* < 0.001, Figure 4, Table 2) for the FSqE_2s_ protocol. No interaction was found between both eccentric protocols; the only difference was in the results of post hoc tests, and both protocols showed no difference in knee extensor values.

### Hamstring to Quadriceps Changes

The hamstring to quadriceps ratios increased (F_1, 31_ = 4.8, *p* = 0.037) only after the FSqE_2s_ protocol.

## Discussion

The main finding of this study is that the fast FSqE_X_ eccentric protocol did not result in a greater PAPE effect on jumping performance than 2-s eccentric loading in the same muscle pattern, and its effect on the knee and hip muscles was more prominent in the FSqE_2s_ protocol. Therefore, the hypotheses that fast eccentric loading would cause greater PAPE than the FSqE_2s_ protocol and that the type of contraction would not alternate in particular muscle groups comparison, were rejected. This finding does not support the presumption that a CA with high force production at high velocities or a plyometric action should result in greater PAPE (Seitz and Haff, 2016; Seitz et al., 2014). On the other hand, our results confirm that the type of the CA during eccentric loading matters (Bridgeman et al., 2017; Hughes et al., 2016; Ong et al., 2016), especially considering the pattern of activated muscles.

The higher number of significant effects in the FSqE_2s_ protocol and larger reported effect sizes after only eccentric loading (Table 1) may mean that eccentric loading itself could be a good strategy to potentiate without fatigue, which is in contrast with previous findings that a ballistic CA is more effective than a non-ballistic one (Suchomel et al., 2016). This difference may be caused by the type of contraction and by different reciprocal pathways resulting from concentric and eccentric loading (Aagaard, 2018; Sekiguchi et al., 2003). The difference in reciprocal PAPE can also be related to increased knee flexion net force in the FSqE_2s_ protocol and the absence of this PAPE in the FSqE_X_ protocol, where knee flexors and extensors sometimes act as antagonist muscles, yet contribute as agonists during squats and jumps. Since PAPE can be succeeded by antagonist loading (Pisz et al., 2023), the FSqE_2s_ protocol may have the effect of greater quadriceps fatigue and antagonist reciprocal activation of knee flexors.

The simultaneous PAPE of hip extensors and knee flexors in the FSqE_2_ protocol with no PAPE in knee extensors can mean that this CA protocol activates the posterior muscle chain, which is desired for power training and injury prevention. This kind of PAPE is expected in other exercises such as hip thrusts, which can increase peak sprint velocity (Williams et al., 2021). Our study confirms that posterior muscle chain PAPE is present after the FSq CA.

The significant performance increases resulting from front squats in this study ranged in effect size between 0.59 and 0.88, which was expected in power analyses and has been reported previously. However, compared to other eccentric methods, the FSqE_2s_ resulted in a higher effect than flywheel PAPE (ES = 0.24) (Beato et al., 2021) for hamstring isokinetic peak torque and a higher effect than isometric quadriceps contraction for quadriceps peak torque (ES = 0.12). Although our study did not show a significant increase in jump height, there was a significant increase in peak force and the rate of force development with moderate effect size (ES = 0.59–0.76) in both protocols. This notion that a CA does not increase jump height but its force/time curve, has been reported previously in half-back squats (Gołaś et al., 2017) and is considered a performance enhancement because it relates to faster muscle action. Other studies have reported a moderate effect size (ES 0.28–0.44) for squats during ballistic-only action (Suchomel et al., 2016), a moderate effect (ES 0.31–0.39) for accentuated eccentric loading (Bridgeman et al., 2017; Hughes et al., 2016), and a small effect size (ES 0.19) for the eccentric leg press (Ong et al., 2016). Therefore, we consider the SqE_2_ protocol to be one of the most effective eccentric CA exercises to elicit PAPE. On the other hand, a higher effect has been reported for plyometric activities (Seitz and Haff, 2016) or back squats (ES = 1.23) (Esformes and Bampouras, 2013).

The results of this study show that different eccentric action protocols have different PAPE results and different effects on local muscle groups. This confirms previous suggestions that there is a higher local effect of PAPE (Downey et al., 2022; Kolinger et al., 2024; Wong et al., 2020) than the global activation mechanisms, which causes the interchange of central fatigue from high-load complex exercises and the potentiation effect in the neural drive (Gandevia, 2001; Taylor et al., 2006). Consequently, this study adds to the PAPE knowledge that the type and duration of eccentric action distinguish between the PAPE levels for particular prime movers and that the local effect of front squats is higher in hip extensors and flexors than knee extensors. Previous studies clearly show a PAPE effect in knee flexion and extension (Batista et al., 2007; Beato et al., 2020, 2021); however, only one study so far has examined the effects of the eccentric action of knee flexion (Beato et al., 2021), and no study has investigated the effects of hip flexion and extension.

The unresolved question of this study concerns the mechanism of eccentric loading, where the eccentric load has been reported to induce less fatigue than a concentric action (Tsoukos and Bogdanis, 2023), and where eccentric force production might be initiated by dynamic stretching (Araya-Ibacache et al., 2022). This effect cannot be explained by two cross-bridge filament muscle action models, where high unexplained residual force has been reported (Herzog, 2014). One explanation may be a force absorption and reutilization by giant protein titin, accounting for over 95% of the passive force in myofibrils. This protein has multiple mechanical functions, including stabilizing myosin filaments and maintaining the stability of sarcomeres, which influence passive and active muscle properties (Anderson et al., 2010; Herzog, 2014).

The absence of a performance increase effect in CMJ height can be caused by a lack of relative strength (relative front-squat strength 1.00 ± 0.21 kg/body mass) (Seitz and Haff, 2016), which may influence especially the 120 of 1RM eccentric performance. However, we were able to observe significant PAPE in other variables. The PAPE effect was reported to be related to the strength level (Seitz and Haff, 2016), and finding a functional protocol for less strong athletes is still necessary, especially considering that such a protocol has already been established for relatively weak (bench press 0.6 ± 0.1 kg/ body mass) women athletes (Pisz et al., 2023). A limitation of this study may be in the muscle model selection, where our protocol targeted the major prime movers in jumping, but did not evaluate the relationship between hip abductors and adductors strength and power performance (Królikowska et al., 2023a, 2023b). Other limitations are the absence of a control group, using just one CA and intensity, individual PAPE window estimation, and measurement of muscle stiffness changes.

## Conclusions

The main finding of this study is that the front squat CA increases jumping performance by enhancement of hip extensors, where the eccentric type of the conditioning activity changes the level of local prime mover muscle performance. Therefore, coaches should apply the two-second eccentric squat exercise as a conditioning activity if their aim is to enhance the contribution of the posterior hip extension muscle chain to the quadriceps' performance. Coaches may also use the concept of eccentric activation only to enhance concentric power during vertical jumps.

Front squat conditioning activity enhances hip extensors more than knee extensors’ performance, and a two-second eccentric front squat conditioning activity enhances the posterior muscle chain to a greater extent than a fast eccentric-concentric action. The different eccentric types of muscle action during a CA alter the level of local muscle enhancement.

Coaches should consider that: 1) different eccentric types of muscle action during a CA alter the level of local muscle enhancement; 2) front squat conditioning activity enhances hip extensor performance to a greater extent than knee extensors; 3) a two-second eccentric front squat conditioning activity enhances the posterior muscle chain performance more than fast eccentric-concentric activities.
